# A green-lipped mussel prevents rheumatoid arthritis via regulation of inflammatory response and osteoclastogenesis

**DOI:** 10.1371/journal.pone.0280601

**Published:** 2023-01-20

**Authors:** SeungCheon Yang, Hong Ki Min, Jin-Sil Park, Hyun Sik Na, Mi-La Cho, Sung-Hwan Park

**Affiliations:** 1 The Rheumatism Research Center, Catholic Research Institute of Medical Science, The Catholic University of Korea, Seoul, South Korea; 2 Lab of Translational ImmunoMedicine, Catholic Research Institute of Medical Science, College of Medicine, The Catholic University of Korea, Seoul, Republic of Korea; 3 Department of Internal Medicine, Division of Rheumatology, Konkuk University Medical Center, Seoul, Korea; 4 Department of Biomedicine & Health Sciences, College of Medicine, The Catholic University of Korea, Seoul, Republic of Korea; 5 Department of Medical Life Science, College of Medicine, The Catholic University of Korea, Seoul, Republic of Korea; 6 Department of Internal Medicine, Divison of Rheumatology, College of Medicine, Seoul St. Mary’s Hospital, The Catholic University of Korea, Seoul, Republic of Korea; Kansai Medical University: Kansai Ika Daigaku, Institute of Biomedical Science, JAPAN

## Abstract

Rheumatoid arthritis (RA) is a chronic inflammatory disorder characterized by progressive joint destruction. Green-lipped mussel (GLM) has chondro-modulatory and anti-inflammatory properties, but the mechanism underlying the effect of GLM on RA is unclear. To investigate the roles of GLM on the pathogenesis of RA, we examined the effects of GLM in collagen-induced arthritis (CIA) mice and osteoclast differentiation. GLM was orally administrated CIA mice at 3 weeks after chicken type II collagen (CII) immunizations. GLM reduced arthritis severity and the histologic score of CIA mice compared to vehicle. The expression of proinflammatory cytokines (TNF-α, IL-1β, and IL-17) was decreased in the ankle joints of GLM-treated CIA mice. The expression of CD4^+^ IL-17^+^ cells decreased in *ex vivo* splenocytes and the spleens of GLM-treated CIA mice. Moreover, GLM inhibited TRAP^+^ multinucleated cells among mouse bone marrow-derived monocytes/macrophages (BMM), and the expression of osteoclast-related genes in mouse BMMs and human monocytes *in vitro*. These results suggest that GLM has potential as a therapeutic agent that can improve disease by controlling pathologic immune cells and osteoclastogenesis.

## Introduction

Rheumatoid arthritis (RA) is systemic autoimmune-mediated arthritis that eventually causes joint destruction [1]. The prevalence of RA is increasing [2], as is the associated socio-economic burden. Several comorbidities (such as osteoporosis) are increased in patients with RA [3], and the mortality of RA patients is higher than the general population [4]. Therefore, RA management is not limited to controlling arthralgia, but also aims to prevent osteoporosis and other comorbidities. Several conventional synthetic, targeted synthetic, and biologic disease-modifying antirheumatic drugs (DMARDs) are used to treat RA patients [5]. However, some RA patients do not respond to DMARDs or experience side effects [6,7]. Therefore, attempts to discover new therapeutic agents are ongoing [8,9].

Interleukin (IL)–17 is a proinflammatory cytokine implicated in the pathogenesis of RA [10]. Type 17 helper T cells (Th17) produce IL-17 and are involved in the pathogenesis of RA [11,12]. Reducing pathologic cytokines, including IL-17, not only ameliorates the inflammatory response of RA but also regulates osteoclastogenesis because proinflammatory cytokines promote osteoclast differentiation [13,14]. Osteoclast differentiation is increased in RA patients [14], and increased osteoclastogenesis induces not only osteoporosis but also joint destruction [15].

The green-lipped mussel (GLM), also known as New Zealand green-lipped mussel (*Perna canaliculus*), contains long-chain omega-3 polyunsaturated fatty acids, docosahexaenoic acid (DHA), and other minor fatty acids (5, 9, 12, 16-nonadecatetraenoic acid, 5, 9, 12, 15-octadecatetraenoic acid, and 5, 9, 12, 15, 18-heneicosapentaenoic acid) with antioxidant and anti-inflammatory effects [16]. Also, GLM’s contents exert anti-arthritic effects in animal models [17,18]. GLM’s content reduced proinflammatory cytokines and reactive oxygen species, and protected chondrocytes [17]. Also, GLM’s content reduced pain and decreased the quantity of analgesic needed by patients with osteoarthritis (OA) [19,20]. However, the effect of GLM in RA has not been assessed.

We investigated the role of GLM using an animal model of RA. We also assessed the effect of GLM on immune cells and osteoclastogenesis.

## Materials and methods

### Animals

Seven-week-old male DBA/1 and C57BL/6 mice were used in this study (Orient Bio Inc., South Korea). The mice were maintained under specific pathogen-free conditions at the Institute of Medical Science of the Catholic University of Korea. They were fed standard mouse chow (Ralston Purina, USA) and housed under controlled temperature (21–22°C) and light (12/12 h light/dark cycle) conditions. All experimental procedures were approved by the Institutional Animal Care and Use Committee of the Department of Laboratory Animals of the School of Medicine, Catholic University of Korea (approval number CUMC 2020-0228-01).

### Collagen-induced arthritis (CIA) induction and GLM treatment

To induce arthritis, chicken type II collagen (CII; Chondrex, USA) was dissolved overnight in 0.1 N acetic acid (4 mg/mL) with gentle rotation at 4°C. DBA/1 mice were injected intradermally (into the tail) with 100 μg of CII emulsified in complete Freund’s adjuvant (Chondrex). Two weeks later, 100 μg of CII, dissolved and emulsified at 1:1 with incomplete Freund’s adjuvant (Chondrex), was administered into the tail dermis as a booster injection. The mice were divided into three groups (5 mice per group). To assess the effect of GLM on the development of CIA, mice were daily orally administrated 200 μL of vehicle (corn oil) only, methotrexate (MTX, 5 mg/kg), or GLM (500 mg/kg) at 3 weeks after the first immunization. GLM powder was provided by The Food Industry Promotional Agency of Korea. GLM was dissolved in corn oil and MTX was dissolved in 0.1% DMSO in phosphate-buffered saline (PBS).

### Clinical assessment of arthritis

Arthritis severity was evaluated twice a week up to 10 weeks after the first immunization to determine the onset and severity of joint inflammation. The severity of arthritis was assessed on a 5-point scale as described previously [21] (0  =  no edema or swelling; 1  =  slight edema with erythema limited to the foot or ankle; 2  =  slight edema with erythema from the ankle to the tarsal bone; 3  =  moderate edema with erythema from the ankle to the tarsal bone; and 4  =  severe edema with erythema from the ankle to the entire leg). The arthritis score of each mouse was calculated as the sum of the scores of the four limbs; the highest possible arthritis score for each mouse was 16. The mean arthritis index was compared between the control and experimental groups.

### Histological analysis and immunohistochemistry

At 10 weeks after the first immunization, mice were euthanized and tissue was obtained. Joint tissue was fixed in 10% (v/v) neutral-buffered formalin (Sigma-Aldrich, USA) and embedded in paraffin. Joint sections (5 μm) were prepared and stained with hematoxylin/eosin and Safranin O to detect proteoglycans. Inflammation was scored as follows: 0, no inflammation; 1, slight thickening of the lining layer or some infiltrating cells in the sublining layer; 2, slight thickening of the lining layer plus some infiltrating cells in the sublining layer; 3, thickening of the lining layer, influx of cells into the sublining layer, and presence of cells in the synovial space; and 4, synovium highly infiltrated with many inflammatory cells. Cartilage erosion was scored as follows: 0, no destruction; 1, minimal erosion limited to single spots; 2, slight-to-moderate erosion in a limited area; 3, extended erosions; and 4, general destruction.

Joint sections were deparaffinized using xylene and dehydrated in an alcohol series. At least five sections from each joint were analyzed. The sections were treated with proteinase K in Tris-ethylenediaminetetraacetic acid buffer for antigen retrieval and washed in 1× PBS (pH 7.5). Endogenous peroxidase activity was quenched with methanol and 3% H_2_O_2_. Immunohistochemistry was performed using the Envision Detection System (Dako, Denmark). The tissues were incubated with primary anti-tumor necrosis factor-α (TNF-α), -IL-1β, -IL-6, -IL-17, and -TRAP (Abcam, UK) antibodies overnight at 4°C, followed by a horseradish peroxidase-conjugated secondary antibody for 30 min. The final colored product was developed using 3,3-diaminobenzidine (Dako, Denmark). The sections were counterstained with Mayer’s hematoxylin and examined by photomicroscopy (Olympus, Japan). Positive areas were analyzed using ImageJ software (NIH, USA).

### Confocal microscopy

Spleen tissues were snap-frozen in liquid nitrogen and stored at −70°C. Spleen tissue sections (5 μm) were fixed in acetone, blocked with 10% goat serum at room temperature for 30 min, and reacted with PE-conjugated anti-IL-17 (eBioscience, USA) and FITC-conjugated anti-CD4 (eBioscience) antibodies. After incubation at 4°C overnight, images were captured using a confocal microscope (LSM 700; Carl Zeiss, Germany). Co-localization was analyzed using ImageJ software.

### Flow cytometry

Spleens were removed from mice and single cells were isolated. Prior to intracellular staining, cells were stimulated for 4 h with phorbol 12-myristate 13-acetate (PMA) (25 ng/mL) and ionomycin (250 ng/mL) in the presence of GolgiStop™ (BD Biosciences, USA). Immunostaining was conducted using fluorescently conjugated antibodies against PerCP-Cy5.5-conjugated anti-CD4 (eBioscience) and FITC-conjugated anti-IL-17 (eBioscience). Intracellular staining was performed using a BD Cytofix/Cytoperm Plus Fixation/Permeabilization Kit (BD Biosciences). Flow cytometry was performed using a CytoFLEX Flow Cytometer (Beckman Coulter, USA).

### Osteoclast differentiation

Bone marrow cells from normal C57BL/6 mouse tibias and femurs were cultured in alpha-minimal essential medium (α-MEM) (Invitrogen, USA) containing antibiotics (Gibco, USA) and 10% heat-inactivated fetal bovine serum (FBS) for 4 h to separate floating and adherent cells. Non-adherent cells were collected and seeded into 48-well plates (Nunc, Denmark), and pre-osteoclasts were cultured in the presence of 10 ng/mL macrophage colony-stimulating factor (M-CSF) (PeproTech, United UK) for 3 days. To generate osteoclasts, cells were cultured with 10 ng/mL M-CSF and 50 ng/mL receptor activator of nuclear factor kappa-Β (RANK) ligand (RANKL) (PeproTech), in the presence or absence of GLM (100 μg/mL) or MTX (10 nM). The medium containing M-CSF, RANKL, and GLM or MTX was changed every 2 days. Osteoclasts were generated after 4–6 days.

Peripheral blood mononuclear cells (PBMCs) from healthy humans were separated from buffy coats using standard density gradient centrifugation over Ficoll–Paque (GE Healthcare Biosciences, Sweden). Cells were seeded into 24-well plates (Nunc) and incubated in 10% α-MEM for 2 h to separate suspended and adherent cells. Adherent cells were cultured with M-CSF 100 ng/mL for 3 days. To generate osteoclasts, cells were cultured with M-CSF 10 ng/mL and RANKL 30 ng/mL, in the presence or absence of GLM (100 μg/mL) or MTX (10 nM). The medium containing M-CSF, RANKL, and GLM or MTX was changed every 3 days. Osteoclasts were generated after 8–10 days. The human experimental procedure was approved by the Institutional Review Board of Seoul St. Mary’s Hospital and written informed consent was obtained (approval number KC17MNSI0405).

### Tartrate-resistant acid phosphatase (TRAP) staining

The TRAP and ALP Double-Stain Kit (TaKaRa, Japan) was used according to the manufacturer’s instructions. Nuclear staining was not performed. Tartrate-resistant acid phosphatase (TRAP)-positive multinuclear cells (MNCs) containing three or more nuclei were counted as osteoclasts.

### Real-time polymerase chain reaction

RNA was extracted using TRI Reagent (Molecular Research Center, USA) and cDNA was synthesized using the Dyne First-Strand cDNA Synthesis Kit (Dyne Bio, South Korea) according to the manufacturer’s protocol. cDNA samples were quantified using the StepOnePlus™ Real-Time PCR system (Applied Biosystems, USA) with SensiFAST SYBR Hi-ROX (Bioline, UK). The following primers were used to amplify mouse genes: *TRAP (Acp5)*, 5′-TCCTGGCTCAAAAAGCAGTT-3′ (sense) and 5′-ACATAGCCCACACCGTTCTC-3′ (antisense); *cathepsin K (Ctsk)*, 5′-CAGCAGAGGTGTGTACTATG-3′ (sense) and 5′-GCGTTGTTCTTATTCCGAGC−3′ (antisense); *Carbonic anhydrase (CA)*, 5′-TGGTTCACTGGAACACCAAA-3′ (sense) and 5′-AGCAAGGGTCGAAGTTAGCA-3′ (antisense); *Calcitonin receptor (Calcr)*, 5′-CGGACTTTGACACAGCAGAA-3′ (sense) and 5′-AGCAGCAATCGACAAGGAGT-3′ (antisense); *Nfatc1*, 5′-CTATCGAGTGTTCCCAGCGG-3′ (sense) and 5′-CATCTTCTTCCCGCCGATGA-3′ (antisense); *Mmp9*, 5′-CTGTCCAGACCAAGGGTACAGCCT-3′ (sense) and 5′-GAGGTATAGTGGGACACATAGTGG-3′ (antisense); and *β-actin*, 5′-GAAATCGTGCGTGACATCAAAG−3′ (sense) and 5′-TGTAGTTTCATGGATGCCACAG-3′ (antisense). The following primers were used to amplify human genes: *TRAP (ACP5)*, 5′-GACCACCTTGGCAATGTCTCTG-3′ (sense) and 5′-TGGCTGAGGAAGTCATCTGAGTTG-3′ (antisense); *RANK(TNFRSF11A)*, 5′-GCTCTAACAAATGTGAACCAGGA-3′ (sense) and 5′-GCCTTGCCTGTATCACAAACT-3′ (antisense); *Cathepsin K(CTSK)*, 5′-TGAGGCTTCTCTTGGTGTCCATAC-3′ (sense) and 5′-AAAGGGTGTCATTACTGCGGG-3′ (antisense); *Calcitonin receptor (CALCR)*, 5′-TGGTGCCAACCACTATCCATGC-3′ (sense) and 5′-CACAAGTGCCGCCATGACAG-3′ (antisense); *NFATC1*, 5′-TGTGCCGGAATCCTGAAACTC-3′ (sense) and 5′-GAGCATTCGATGGGGTTGGAG-3′ (antisense); *MMP9*, 5′-CGCAGACATCGTCATCCAGT-3′ (sense) and 5′-GGATTGGCCTTGGAAGATGA-3′ (antisense); and *β-actin*, 5′-GGACTTCGAGCAAGAGATGG-3′ (sense) and 5′-TGTGTTGGGGTACAGGTCTTTG-3′ (antisense). mRNA levels were normalized to that of β-actin.

### Statistical analysis

Statistical analysis was performed using Prism (ver. 5.01; GraphPad Software Inc., USA). We used two-way analysis of variance (ANOVA) (with the Bonferroni *post hoc* test) about the assessment of arthritis to compare arthritis severity and incidence by experimental groups and time. Values are presented as means ± standard error of the mean (SEM). Mann-Whitney test (two-tailed) was used for histology, immunohistochemistry, flow cytometry and confocal compared to control, and values are presented as means ± SEM. Mann-Whitney test (two-tailed) was used for osteoclast differentiation and values are presented as means ± standard error (SD). A value of *p* < 0.05 was regarded as indicative of statistical significance.

## Results

### GLM attenuates CIA development

**Fig 1:**
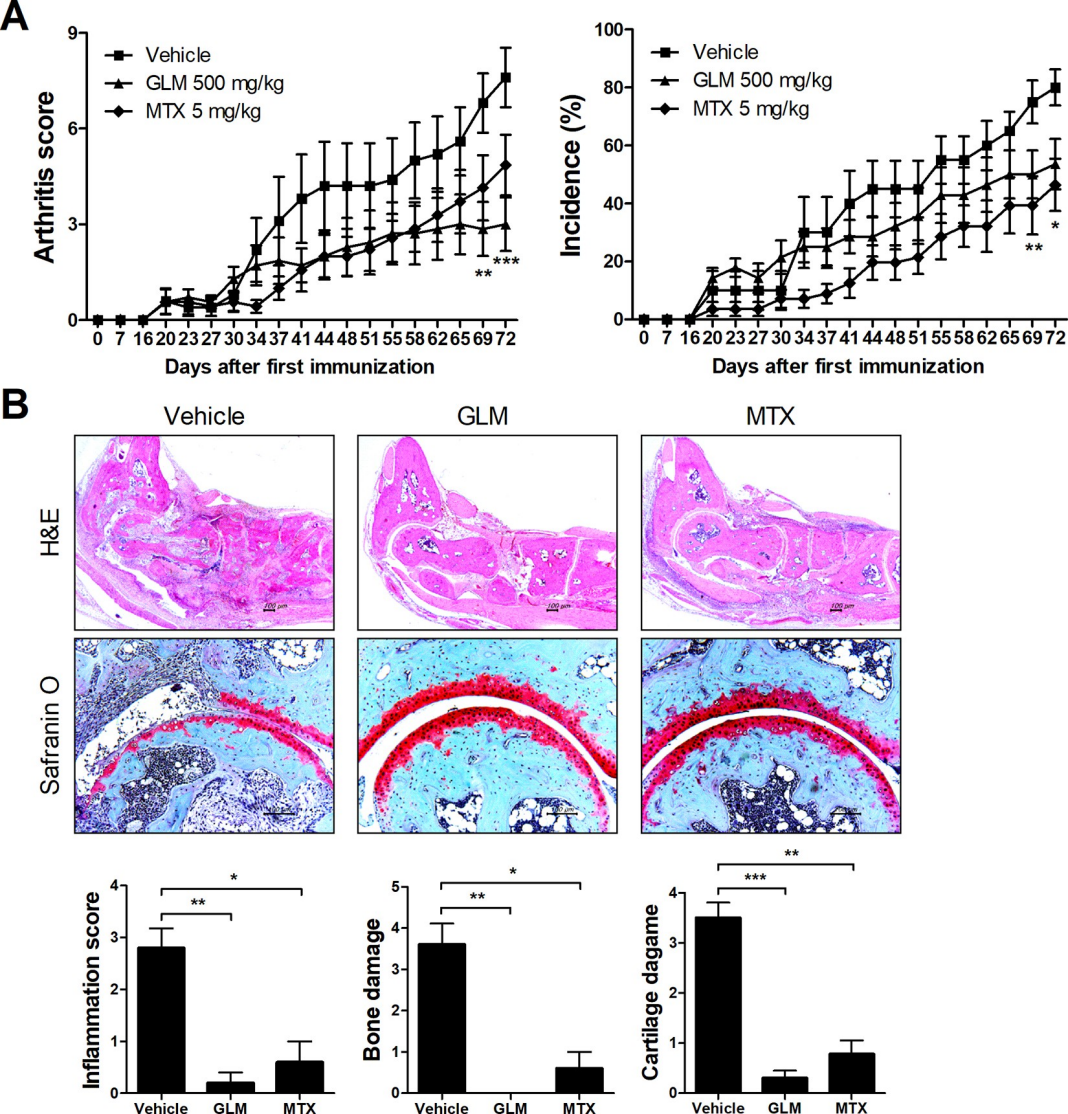
GLM reduced the severity of CIA. Three weeks after the first immunization, CII-induced DBA/1J mice were injected daily with GLM (500 mg/kg) or MTX (5 mg/kg), with oral administration of corn oil (200 μL) serving as a control. (A) Arthritis development was assessed based on the arthritis score and incidence. Representative images from one of two independent experiments are shown. (B) Histological staining of joint tissue sections with hematoxylin/eosin (original magnification, 40×) and safranin O (original magnification, 200×). Representative images are shown. The graphs depict the degree of inflammation, bone damage, and cartilage damage. Data are means ± SEM. *P < 0.05, **P < 0.01, ***P < 0.001.

To examine the effect of GLM on the development of CIA, we administered GLM, MTX, or vehicle to CII-induced DBA/1 mice for 7 weeks from 3 weeks after the first immunization. The GLM-treated group had a significantly lower arthritis score compared to the vehicle-treated group, whereas the MTX-treated group did not show a significant difference compared to the vehicle group. There were no significant differences in the incidence of arthritis between GLM-treated and vehicle-treated groups (Fig 1A). At 10 weeks after the first immunization, joints in the GLM- and MTX-treated groups showed significantly attenuated inflammation, bone erosion, and cartilage damage compared to the vehicle-treated group (Fig 1B).

### GLM suppresses the expression of inflammatory cytokines in the joints of CIA mice

**Fig 2:**
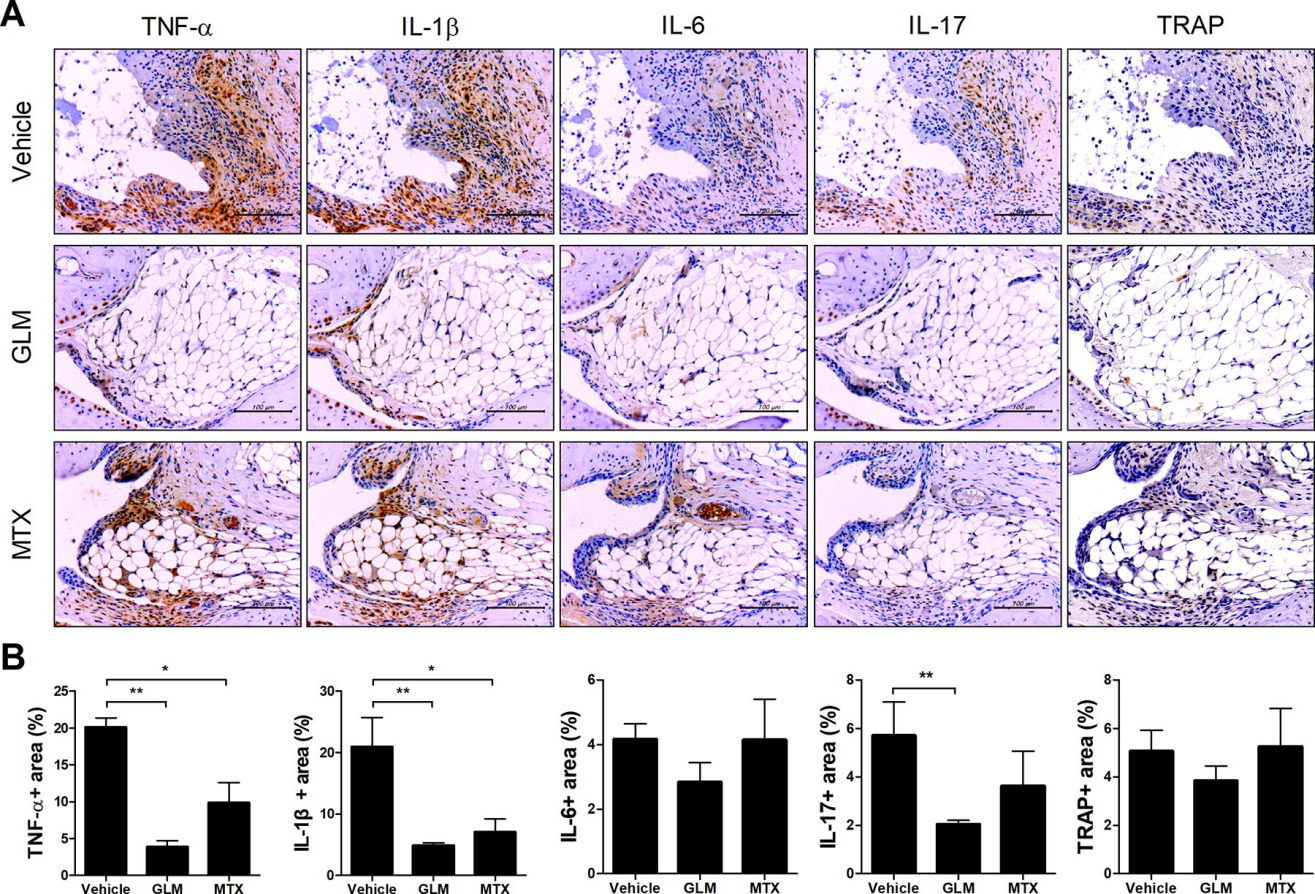
GLM suppressed the expression of inflammatory cytokines in joints. Three weeks after the first immunization, CII-induced DBA/1J mice were injected daily with GLM (500 mg/kg) or MTX (5 mg/kg), or orally administered corn oil (200 μL) as a control. (A) At 10 weeks after the first immunization, the expression levels of TNF-α, IL-1β, IL-6, IL-17, and TRAP in the ankle joints were determined by immunohistochemical staining. Representative images from one of two independent experiments are shown. Bars are percentage positive areas per field. Data are means ± SEM. *P < 0.05, **P < 0.01.

We performed immunohistochemical staining of the joints of GLM-treated CIA mice. The expression of the pro-inflammatory cytokines TNF-α, IL-1β and IL-17 in the joint was significantly decreased in the GLM- compared to the vehicle-treated group (Fig 2A and 2B).

### GLM suppresses Th17 differentiation

**Fig 3:**
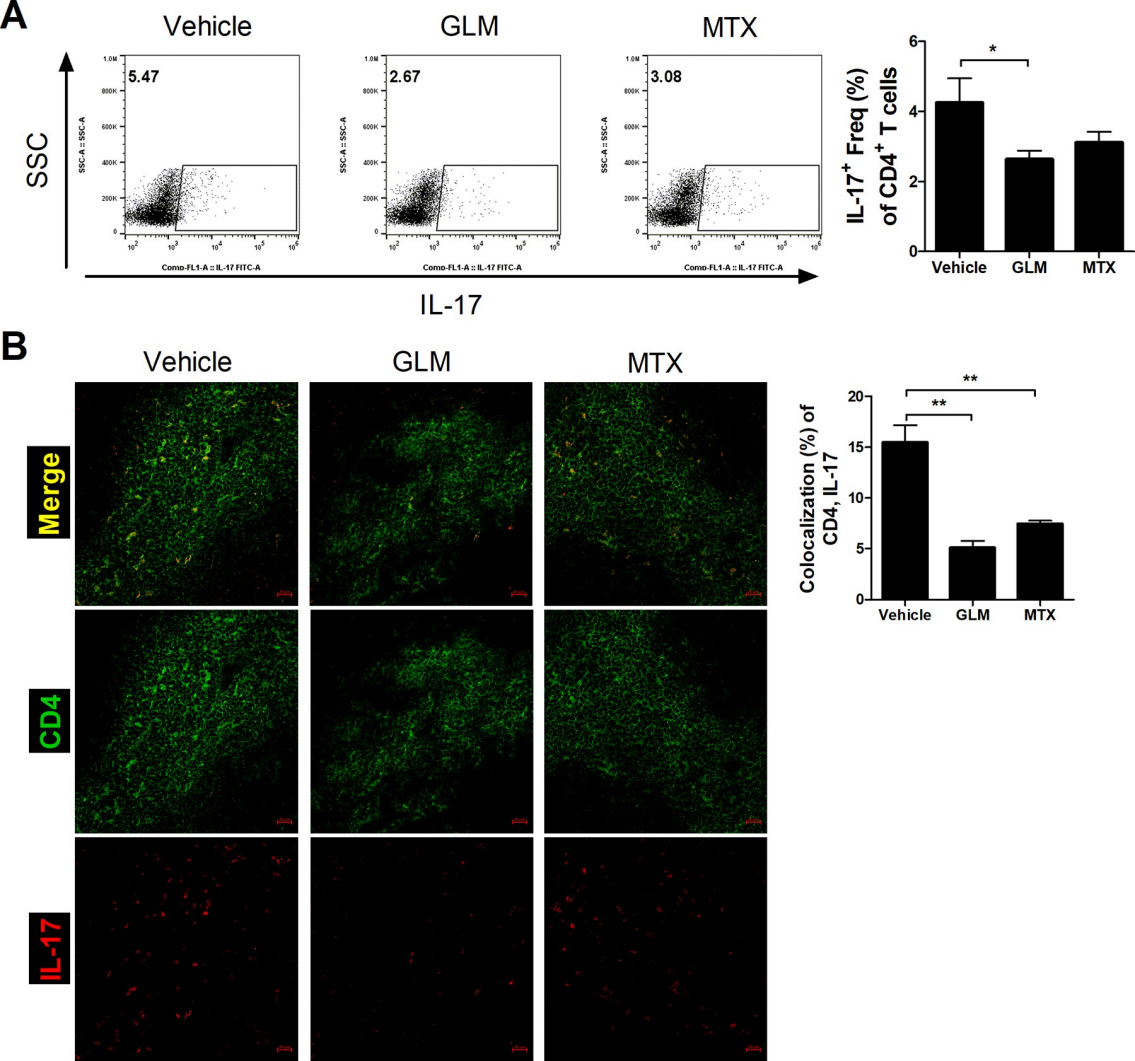
GLM suppressed the Th17 proliferation. Three weeks after the first immunization, CII-induced DBA/1J mice were injected daily with GLM (500 mg/kg) or MTX (5 mg/kg), or orally administered corn oil (200 μL) as a control. (A) At 10 weeks after the first immunization, the number of CD4+ IL-17+ cells in *ex vivo* splenocytes was analyzed by flow cytometry. Representative flow cytometry plots from one of two independent experiments are shown. (B) Confocal images of CD4+ IL-17+ cells in the spleen. Bars are percentages of co-localization of CD4 (green) and IL-17 (red). Data are means ± SEM. *P < 0.05, **P < 0.01.

We next investigated the number of Th17 cells in the spleen of CIA mice by flow cytometry. The population of CD4^+^ IL-17^+^ cells (Th17) in splenocytes from the GLM-treated group was significantly reduced compared to the vehicle-treated group (Fig 3A). CD4^+^ IL-17^+^ cells in the spleens of CIA mice were analyzed by confocal microscopy. The number of Th17 cells was significantly decreased in the GLM-treated group compared to the vehicle-treated group (Fig 3B).

### GLM inhibits osteoclast differentiation in mouse and human

**Fig 4:**
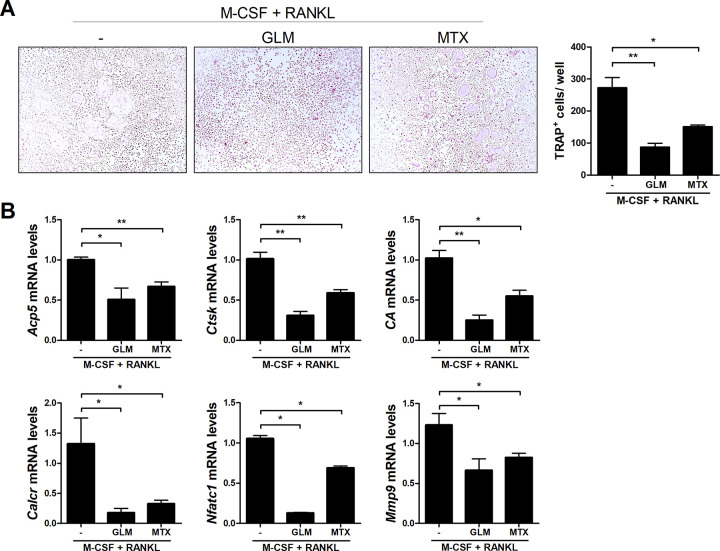
Effect of GLM on the osteoclastogenesis of mouse cells. Bone marrow-derived monocytes/macrophages from normal C57BL/6 mice were cultured with M-CSF (10 ng/mL) and RANKL (50 ng/mL). (A) After 5 days, cells were stained for TRAP. Representative images from one of two independent experiments are shown. The number of multinucleated TRAP+ cells was determined. (B) mRNA levels of *TRAP*, *cathepsin K*, *carbonic anhydrase*, *calcitonin receptor*, *NFATC1*, and *MMP9* determined by real-time PCR. Data are means ± SD. *P < 0.05, **P < 0.01.

To investigate the effect of GLM on *in vitro* osteoclastogenesis, bone marrow-derived monocytes/macrophages (BMMs) from normal C57BL/6 mice were stimulated with M-CSF and RANKL in the presence or absence of GLM (100 μg/mL) or MTX (10 nM). Counts of TRAP^+^ MNCs were significantly decreased in GLM-treated cells compared to the control (Fig 4A). The mRNA levels of the osteoclastogenesis associated genes *Acp5*, *Ctsk*, *CA*, *Calcr*, *Nfatc1*, and *Mmp9* were significantly reduced in GLM-treated cells (Fig 4B).

**Fig 5:**
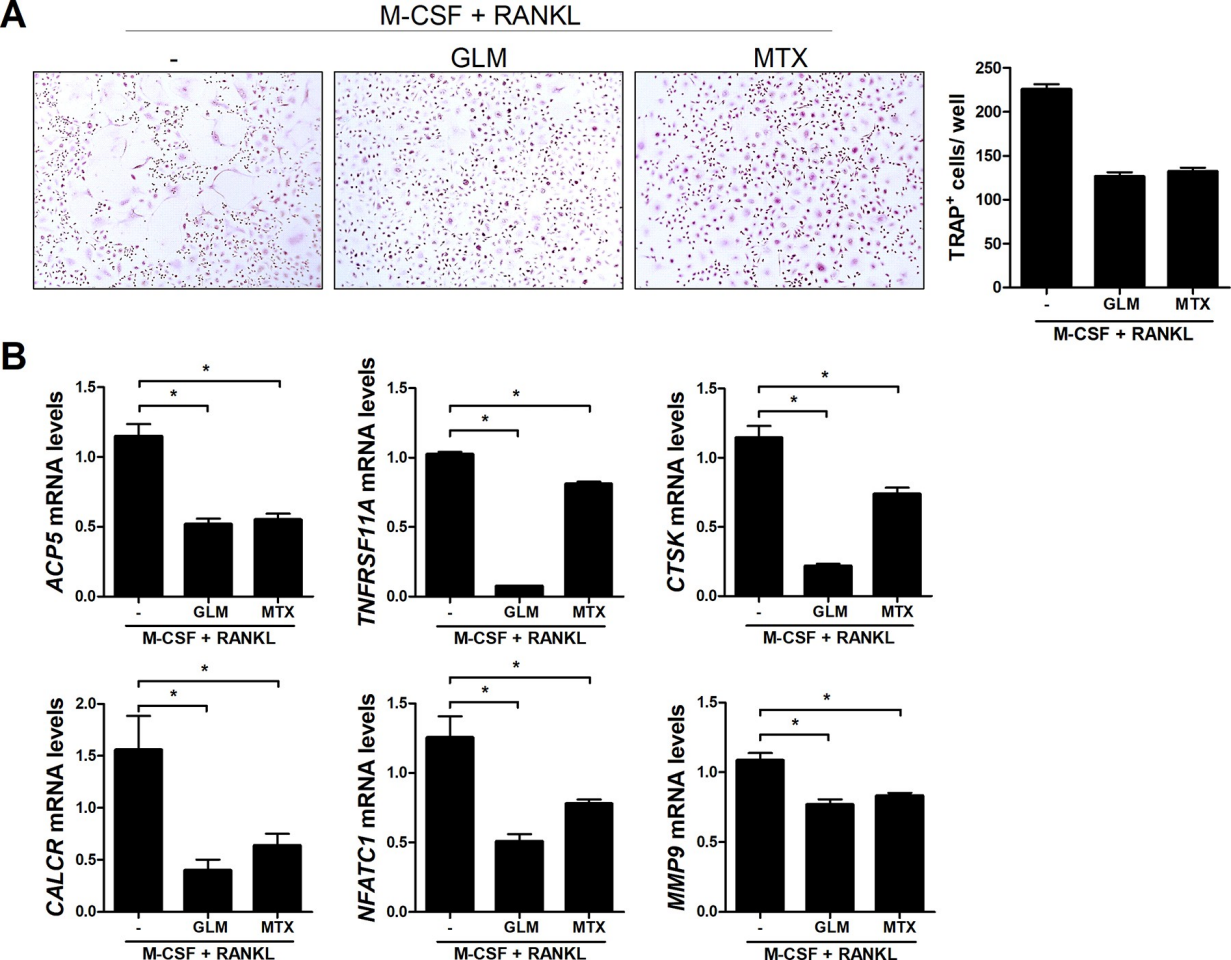
Effect of GLM on osteoclastogenesis in human cells. Human PBMC-derived monocytes were cultured with M-CSF (10 ng/mL) and RANKL (30 ng/mL). (A) After 10 days, cells were stained for TRAP. Representative images from one of two independent experiments are shown. The number of multinucleated TRAP+ cells was determined. (B) mRNA levels of *TRAP*, *RANK*, *cathepsin K*, *calcitonin receptor*, *NFATC1*, and *MMP9* determined by real-time PCR. Data are means ± SD. *P < 0.05.

Next, PBMC-derived monocytes were cultured with M-CSF and RANKL in the presence or absence of GLM (100 μg/mL) or MTX (10 nM). GLM non-significantly inhibited TRAP^+^ MNCs formation (Fig 5A). The mRNA levels of the osteoclastogenic markers *ACP5*, *TNFRSF11A*, *CTSK*, *CALCR*, *NFATC1*, and *MMP9* were significantly decreased in GLM-treated cells compared to the control (Fig 5B).

## Discussion

GLM exerted an anti-arthritis effect in CIA mice. Also, GLM suppressed osteoclastogenesis *in vitro*. These effects of GLM were comparable with MTX, which is the most frequently used DMARD for RA.

GLM contains abundant long-chain omega-3 polyunsaturated fatty acid, among other fatty acids [16]. The therapeutic role of GLM was first investigated in OA. GLM in OA mice reduced nociception and the expression of proinflammatory cytokines (IL-1β, IL-6) and inducible nitric oxide synthase. It also exerted a chondroprotective effect by decreasing the production of chondrocyte-degrading enzymes (matrix metalloproteinase-3/13, ADAMTS5), and suppressing chondrocyte necroptosis [17]. These anti-arthritis/anti-inflammatory effects of GLM are comparable to the nonsteroidal anti-inflammatory drug (NSAID) celecoxib, which is frequently used for OA [17]. Also, a clinical trial suggested that GLM may reduce pain and functional capacity in OA patients [19]. Another clinical trial did not meet its primary endpoint but showed improvements of joint stiffness and less painkiller use in the GLM-treated group [20]. In this study, the expression levels of proinflammatory cytokines (TNF-α, IL-1β, IL-17) were decreased by GLM in the joints of CIA mice, similar to methotrexate. In addition, IL-17–producing CD4^+^ T cells were suppressed by GLM.

The prognosis of RA depends on the degree of joint destruction, because joint destruction in RA is progressive and irreversible [22]. Prevention of joint destruction is a major treatment goal in RA, and synthetic and biologic DMARDs aim to not only control arthritis but also slow joint destruction [5,23]. Osteoclasts are the main effector cells in joint destruction [15], and increased proinflammatory cytokines in RA promote their differentiation [14]. In this study, GLM reduced the osteoclastogenesis of mouse- and human-derived osteoclast precursor cells. GLM suppresses osteoclastogenesis *in vitro* and reduced the production of the proinflammatory cytokines (TNF-α, IL-1β, and IL-17) that increase osteoclast differentiation [13,14]. Therefore, GLM may preserve joint structure by directly inhibiting osteoclastogenesis and reducing the production of osteoclast-promoting cytokines.

This study was performed using an animal model of RA, and *in vitro*; the *in vivo* effect of GLM in patients with RA was not evaluated. Also, the effect of GLM was compared with MTX but not biologic DMARDs. Therefore, we cannot conclude that GLM can serve as an RA treatment option. However, GLM regulated pathologic immune cells, reduced the production of proinflammatory cytokines, and suppressed osteoclastogenesis. Therefore, GLM has potential as an anti-arthritic; a further *in vivo* study of GLM in RA patients is needed to validate our findings.

In general, women are more frequently affected than men in the development of autoimmune diseases, including RA [24]. But we used only male mice for the CIA study, as the incidence of arthritis is somewhat higher in male than in female mice [25]. There is a limitation in that the sex of the animal model differs from that of human RA, despite CIA being used because it shares many similar pathological and immunological features with human RA. Further research is needed on the effect of GLM according to gender.

In conclusion, GLM prevented arthritis in CIA mice, accompanied by a reduction in proinflammatory cytokine expression. GLM also reduced the number of IL-17–producing CD4^+^ T cells and suppressed osteoclast formation. Therefore, GLM may have therapeutic potential for RA.
